# Parents’ received and expected information about their child’s radiation exposure during radiographic examinations

**DOI:** 10.1007/s00247-018-4300-z

**Published:** 2018-11-13

**Authors:** Heljä T. Oikarinen, Anne M. Perttu, Helena M. Mahajan, Leila H. Ukkola, Osmo A. Tervonen, Aino-Liisa I. Jussila, Anja O. Henner

**Affiliations:** 1 Department of Diagnostic Radiology, Oulu University Hospital, POB 50, 90029 OYS Oulu, Finland; 2 Oulu University of Applied Sciences, Kiviharjuntie 4, 90220 Oulu, Finland

**Keywords:** Children, Communication, Informed consent, Ionising radiation, Radiography

## Abstract

**Background:**

Despite regulations, insufficient information is provided to adult patients prior to their radiologic examinations. Information regarding paediatric patients has not been systematically studied.

**Objective:**

To survey parents’ experience and wishes for information in connection with their child’s radiographic examination.

**Materials and methods:**

We provided a questionnaire to consenting parents of children younger than 12 years old at a university hospital. The questionnaire asked parents about the information obtained from the referrer prior to the radiograph, the chance to discuss with the referrer and their wishes regarding future information. Forty-one parents responded to the survey. Twenty-five children were referred for radiography of extremities, the others for dental, body and skull examinations.

**Results:**

Altogether 34/41 (83%, 95% confidence interval [CI] 69–91%) parents said they received adequate information on the purpose of the examination, 8/35 (23%, 95% CI 12–39%) on other options and 3/41 (7%, 95% CI 3–19%) on radiation dose. Ten of 41 parents (24%, 95% CI 12–40%) said they were aware of radiation exposure. The number of previous radiology examinations was not sufficiently discussed. The communication was scored as mean 6.5 (95% CI 5.8–7.1) on a scale from 4 (poor) to 10 (excellent). Thirty-eight of 40 (95%, 95% CI 84–99%) of parents expected information on the purpose, 35/40 (88%, 95% CI 74–95%) on radiation dose and 31/40 (78%, 95% CI 63–88%) on other options. Symbols of radiation and corresponding period of natural background radiation are preferred to convey the dose. A referrer is the preferred source of information.

**Conclusion:**

Parents did not feel adequately informed prior to their child’s radiographic examination. Parents expect more information about the purpose, dose and alternative tests.

## Introduction

It is the responsibility of the referrer as well as the practitioner to perform justification of examinations exposing children to ionising radiation. However the patient’s opinion should also be considered in the justification process, i.e. the child/parents should be provided with due information before consent is obtained [1, 2]. Informing patients on the possible risks of ionising radiation has already been required [3], and European Union Council Directive 2013/59 underlines information on the benefits and the risks [2]. Furthermore, according to the legislation regarding patient rights, patients should be informed about the benefit and possible risks of a treatment as well as about other options [4]. Regarding paediatric imaging, parents or caregivers should thus be informed about the benefits and risks of the examination even without asking. Health care providers requesting or performing procedures have a shared responsibility to communicate with the patients [5].

Approximately 4 million radiology examinations per year (excluding dental X-ray examinations) are performed in Finland and about 7% of these are performed on children. Eighty-five percent of the total are plain radiographs, whereas in children 96% of radiology examinations are radiographs [6]. Although the radiation dose of a single radiographic examination is usually small, the total number of these examinations is high.

There has been suspicion that little or no information about planned examination and radiation is provided to patients [1, 7–9]. There is also insufficient knowledge regarding radiation not only among patients and parents, but also among practitioners and referrers, including paediatricians, yet some increase in awareness and communication has recently been found [10–16]. Our previous study showed that adults expect to receive diverse information including information on the dose and risks of radiation [17]. Concerning paediatric patients, some publications have concentrated on risk information in connection with CT examinations [18, 19]. Radiation protection efforts have recently been globally focused, particularly on children [5, 20]. Paediatric CT, with its increasing use and higher dose, has been one of the main concerns [21, 22]. However, due information should be provided in connection with plain radiographs as well — it is important to inform and reassure parents of the low doses and minimal possible risks related to justified radiographs.

The purpose of this study was to find out parents’ experience regarding information obtained from the referrer prior to their child’s plain radiographic examination at a university hospital. In addition, we enquired about parents’ wishes for information received in the future.

## Materials and methods

The study was performed May–June 2013 in the Department of Diagnostic Radiology of Oulu University Hospital (population base about 400,000). The study was approved by the institutional review board and oral informed consent was required. The paediatric radiology unit has three paediatric radiologists and 15 radiographers. About 23,000 radiographs and about 4,200 ultrasound, 400 CT and 2,300 MRI examinations are performed annually on children in different units of the radiology department.

**Fig. 1:**
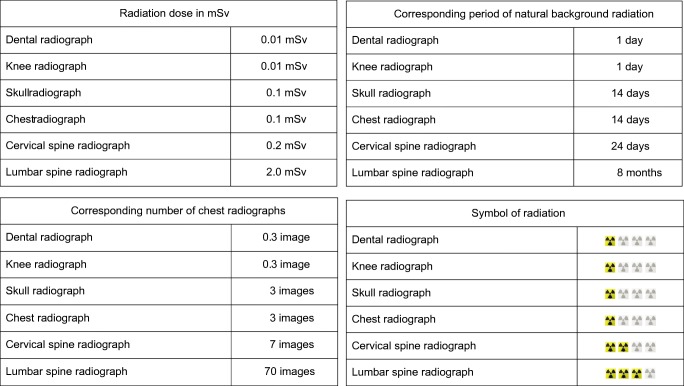
The table included in the questionnaire demonstrates four forms to convey the dose of radiation. The parents were able to choose one or many options

We prepared a questionnaire for parents enquiring about information provided by the referrer prior to their child’s present radiographic examination (Table 1). We also questioned whether parents had the opportunity to discuss with the referrer. Furthermore, there were questions regarding wishes for communication in the future (Table 2). To find out preferences concerning how to convey the dose of radiation, we prepared a table to demonstrate four forms of information (Fig. 1). We included the table in the questionnaire and the parents were able to see all the forms at once and to choose one or many options. In addition, demographic data were requested, and parents were asked to estimate the number of previous radiology examinations performed on their child.

**Table 1:** Questions regarding the information and communication in connection with present examination and type of queries

1. Whether parents received enough information regarding the following issues from the referrer and whether it was understandable (5-steps Likert scale questions) • purpose of the examination • dose of radiation • other possible options (e.g., ultrasonography, MRI)	
2. Whether there was a chance to discuss the following issues with the referrer (5-steps Likert scale questions) • symptoms of the child • purpose of the examination / dose of radiation / other possible options • number of previous radiology examinations	
3. Overall source of information regarding radiation use (multiple answer multiple-choice question) • oral from the staff, written from the hospital, both oral and written, other (how?), or none	
4. Grading the overall information and communication, Likert scale from 4 (poor) to 10 (excellent). (Similar scale is used for grading in schools in Finland)

**Table 2:** Questions regarding the wishes for future information related to radiographs and type of queries

1. Wishes related to information provided by a referrer regarding the following issues (5-steps Likert scale questions) • purpose of the examination • dose of radiation • other possible options	
2. Source of information (multiple answer multiple-choice question) • referrer, radiographer, someone else (who?), no one	
3. Method of communication regarding the purpose, dose and other possible options (single answer multiple-choice question) • oral, written, both, none	
4. Method of conveying dose of radiation (multiple answer multiple-choice question, see Fig. 1)	
5. Spontaneous wishes for the content of information (open question)

The questionnaire was distributed in the paediatric, emergency and dental units of the radiology department. It was provided with a cover letter to parents of paediatric patients up to 12 years of age just before or after the radiographic examination. Hence only children whose radiographs were performed in the radiology units were included. Radiographers and radiographer students who recruited participants were informed about the study in advance, and they were given written guidelines on how to provide the questionnaire to parents. The questionnaire and the cover letter were made easy to understand. The validity was tested with 10 persons who were not professionally familiar with radiographic examinations.

The method was based on convenience sampling [23]. The aim was to include children undergoing various plain radiographs. The selection of the parents was otherwise coincidental, depending on, for example, scheduling. Only parents who were present when the referrer was considering the examination were included in the study; other accompanying adults were excluded because we wanted to focus on parents only. The cover letter stated that the referrer had judged the present examination to be necessary. It included information on the survey as well as contact information, and it stated that participation was voluntary, confidential and anonymous.

In the case of consenting parents, the staff first filled in the type of the present examination and the referring unit. The parents themselves filled in the questionnaire in the waiting room and returned it to the return box. We classified the present radiographs into five categories according to the average dose of radiation (Table 3).

**Table 3:** The age groups of the children, the reported number of previous radiology examinations performed on the children, and the present examinations the children had been referred for

Age groups (years)	*n*	Number of previous radiology examinations	*n*	Present radiograph		*n*
0–3	4	None	10	Small bones	Fingers, hand, wrist, toes, ankle, heel	15
4–6	4	1–5	26	Extremities	Elbow, arm, knee	10
7–9	22	6–10	1	Dental	Panorama tomography, lateral skull	9
10–12	10	≥11	2	Body area	Thorax, shoulder, hip	5
		Not known	1	Skull	Skull	1
Total	40^a^		40^b^			40^c^

^a^Age not reported in one case

^b^Number not reported in one case

^c^Present radiograph not filled in one case

**Fig. 2:**
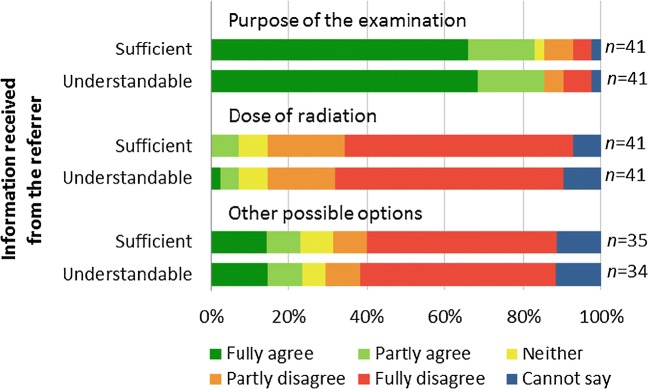
Information received from the referrer (5-steps Likert scale was used). The parents were mostly informed about the purpose of the examination but only seldom about the dose of radiation or other possible options (green indicates positive replies). The information received had been understandable

**Fig. 3:**
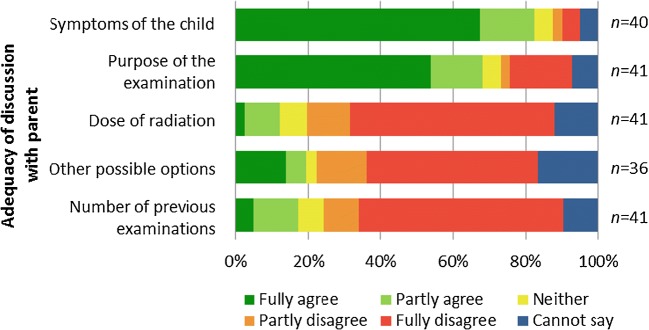
Results regarding the adequacy of discussion with the referrer (5-steps Likert scale was used). There had been most discussion about the symptoms of the child and the purpose of the examination

**Fig. 4:**
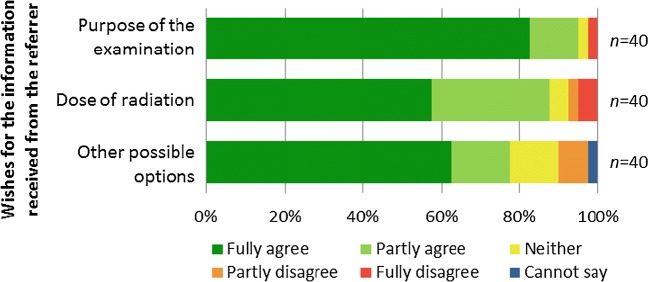
Wishes of the parents regarding information received from the referrer (5-steps Likert scale was used). Most of the parents expect information on the purpose, dose and other possible options

The data were described using frequency distributions and analysed using SPSS Statistics 24 (IBM, Armonk, NY). We analysed the open question using content analysis. While calculating the proportions regarding the 5-steps Likert scale questions, we joined the answers “fully agree” and “partly agree” into “agree”; similarly, we combined the answers “fully disagree” and “partly disagree” into “disagree” (Figs. 2, 3 and 4). The 95% confidence intervals (CI) for the proportions were calculated using the Wilson score method without continuity correction. The mean of the score (with 95% confidence intervals) for the overall communication was also calculated.

## Results

Forty-one parents responded to the survey. Most of the invited parents consented but the proportion of consents or the reason for refusal was not registered. There were 26 women and 15 men. Eighteen of the children were girls and 22 were boys; in one case, age and gender was not filled in. Most of the children were 7–12 years old, and most of the children had 1–5 previous radiology examinations (Table 3). The present examinations are shown in Table 3. There were 21 respondents from the paediatric unit, 11 from the emergency unit and 9 from the dental unit. Twenty-one children had been referred from primary care and 17 from the hospital. The referring unit was not filled in in three cases.

### The information received

Altogether 34/41 (83%, 95% CI 69–91%) of the parents said they had received enough information on the purpose of the examination, 3/41 (7%, 95% CI 3–19%) on the dose of radiation and 8/35 (23%, 95% CI 12–39%) on other options (Fig. 2). The information received had mostly been understandable.

The possibility to discuss the symptoms of the child and the purpose of the examination with the referrer had been good, but there was less discussion on the radiation dose, other imaging options and the number of previous examinations (Fig. 3).

In all, 10/41 (24%) parents were aware of radiation use during the examination. Regarding this information, three parents had obtained oral information from the staff and one parent both oral and written information from the hospital, two had obtained information via Internet and two from a poster in the hospital. Two parents were aware of radiation use because of their previous visits, and one parent “realised it when the child was left by himself with radiation shields on during the examination”. The parents scored the overall communication as mean 6.5 (95% CI 5.8–7.1) on a scale from 4 (poor) to 10 (excellent).

### Wishes for information in the future

Altogether 38/40 (95%, 95% CI 84–99%) parents wished to obtain information on the purpose of the radiograph, 35/40 (88%, 95% CI 74–95%) on the radiation dose and 31/40 (78%, 95% CI 63–88%) on other imaging options (Fig. 4). The preferred sources for the information are the referrer and radiographer (Table 4). Regarding the method of giving information, oral information is preferred. Two parents did not wish for information from any source and three parents did not wish for information given in any way. When preferences to convey the dose were explored, parents preferred symbols of radiation and corresponding period of natural background radiation (Fig. 1 and Table 4).

**Table 4:** The wishes for the source of information and the method of communication regarding plain radiographs and the method of conveying the dose of radiation

Source of information	Method of communication	Communication of dose of radiation
Referrer: 32/38 (84%)	Oral: 18/40 (45%)	Symbols: 16/29 (55%)
Radiographer: 19/38 (50%)	Written: 8/40 (20%)	Natural background rad^a^: 16/29 (55%)
Someone else: 0	Oral and written: 11/40 (28%)	Dose in mSv: 6/29 (21%)
Nobody: 2/38 (5%)	None: 3/40 (8%)	Chest X-ray^b^: 3/29 (10%)

^a^Corresponding period of natural background radiation

^b^Corresponding number of posteroanterior chest X-rays

## Discussion

The importance of patient information concerning the benefits and risks of radiologic examinations has been globally highlighted [7, 24–26]. Some recent studies have shown that adult patients do not receive adequate information in connection with their examinations [8, 9]. To our knowledge, this is the first survey to assess parents’ experience of obtaining information in connection with their child’s examination. The study revealed that parents are not sufficiently informed, either. Specifically, information regarding radiation is poor. Furthermore, parents wish to obtain diverse information even in connection with radiographic examinations.

The study population of the present study comprised parents of children of 0–12 years old. This age group was chosen because parents alone are usually more responsible for the treatment of the child up to the age of 12 years than with older children. The survey concentrated on radiographs only. They make up most of the examinations in children and they can be repeatedly performed, for example, in the case of premature babies or children with scoliosis. We realise that CT could be the main preoccupation. However, we did not want to cause anxiety; because the study was performed using a questionnaire we wanted to focus on examinations that expose children to a low radiation dose. In addition, the number of paediatric CT scans in Finland has recently been decreasing; 1.2% of them were performed on children in 2015 [6]. Furthermore, appropriate information is required in connection with radiographs as well.

Parents were mostly informed about the purpose of the examination. However they obtained little information on other options; even when there are no other options, this point could be mentioned. Furthermore, only 7% of the parents received enough information on radiation dose. Parental involvement was also insufficient. We realise that the parents might have received some information from the staff of the radiology department or in a letter from the hospital. In fact, 24% of the parents were aware of radiation use during the examination. However, only four parents had obtained this information from the hospital staff or an information letter. Therefore, it is probable that most of the information, if any, had been received from the referrer. The practice of posting an information letter is also variable at the hospital concerned.

Another study regarding children that was conducted among staff also found that the referrer or the staff of the radiology department explained the purpose while less information was given about the risks or options [16]. There is also a report of information in connection with imaging in a cancer care setting. Most of the participants, including three parents of children with malignancy, reported that the referrer did not initiate benefit–risk discussion [9].

Our previous study assessed information received by adult patients who had undergone examinations with various dose levels [8]. The referrer had mostly given information on the purpose of the examination. Both our present and previous studies reveal insufficient information regarding radiation. It is likely that the lack of practical guidelines has decreased staff’s willingness to provide information at the hospital concerned. Uncertainty, fear of causing anxiety, and haste might also reduce the willingness to communicate more information about the examination.

According to the present study, parents wish to receive diverse information even in connection with radiographic examinations. Almost 90% expect to obtain information on the dose of radiation. According to the open question, some parents would also like to talk about the effects, detriment and dangers of radiation. Regarding our survey, we enquired about the dose instead of the risks. It would be appropriate to interview parents in person to find out their opinions regarding risk information; it would be more comfortable, and parents would have the possibility to ask questions.

Parents preferred symbols of radiation and the corresponding period of natural background radiation to convey the dose. The latter and corresponding number of chest radiographs are often recommended [1, 5]. Adult patients preferred symbols and verbal scale [17]. Overall, symbols, verbal scale or comparison to natural background radiation could be understandable to demonstrate the dose. It is possible that symbols of radiation are perceived as symbols of possible risks as well. The table with one to three symbols was developed only for the survey and should be processed further for use in practice regarding different examinations. In addition, the numbers in the table were generalised and the effective dose of some examinations has since reduced. Furthermore, the referrer and radiographer are the preferred sources for information. In fact, parents do not usually meet the radiologist as often as the other staff members.

According to other studies, patients expect information on radiation dose and risks, the rationale for the planned examination and other options. Active participation in decision-making is also appreciated [9, 17]. Referrer and readily available reference material are the preferred sources. Furthermore, most parents wished to be informed of lifetime malignancy risk from head CT performed on their child [19].

Consequently, we should respond to the wishes and not be too concerned about causing anxiety. In fact, appropriate information might even reduce anxiety. However the extent of the information should depend on the nature of the examination and on the recipient [1, 27–29]. Efficient communication is a challenging task with difficult understanding of the dose and risks and uncertainty of the risks [30]. Feelings and personal circumstances of the recipient must be accounted for, as well. In general, the referrer has the most complete clinical data and could discuss with the parents while considering a request for an examination. A radiographer or radiologist could give additional information if needed. Parents should always have the opportunity to ask questions. Overall, both oral and written information could be provided [17]. In the case of radiographs, concise written information might be the easiest way to inform adult patients. However, parents of paediatric patients seem to appreciate oral information even in connection with plain radiographs. Nevertheless, the scheme might differ among countries. For referrers and practitioners, a national registry revealing previous examinations and cumulative dose would be useful.

Some guidance regarding patient information has recently been published [1, 5, 31]. The World Health Organization (WHO) guidelines focus on benefit–risk communication in paediatric imaging [5]. The present study is a part of our research and developmental project regarding information in connection with different radiology examinations. Our studies have revealed a need for better information regarding both adult and paediatric patients. We realise that practical guidelines, easy access to information, and education are needed. So far, concise educational information for the staff, and separately for the patients, has been drawn up. Based on this material, video clips for patients have also been prepared. The material will soon be displayed in the virtual hospital to be broadly available in Finland. We believe that digital material could, at least partly, replace oral information and facilitate the challenging task. Clear guidelines are also being prepared in our area regarding the extent of information and the responsibilities for providing information.

There are some limitations in this study. The study is from one hospital and it focused on radiographs only. However, to our knowledge, this study is the first to assess both the experiences and wishes of parents, and it might reflect the situation elsewhere as well. It can also be assumed that while parents wish for oral information regarding radiographs, they expect to receive information regarding all examinations. Similar studies concerning, for example, CT might require a personal interview with the parents. The survey is also from some years ago. However, it is a part of a larger information project. The selection of the parents might also cause a risk of bias. Furthermore, the aim was to recruit as many parents as reasonably possible, depending on scheduling, during the 2-month study period. However, apart from some refusals, there was some negligence of recruitment. Therefore, the number of the participants is limited. Finally, it is possible that the parents had received more information than they were able to remember. Some questions might also have been unclear because not all the questions were answered by all parents.

## Conclusion

Parents expressed that they are insufficiently informed in connection with their child’s radiographic examination. Specifically, information about radiation exposure was thought to be insufficient. Symbols of radiation and the corresponding period of natural background radiation were preferred to convey the magnitude of the radiation dose.
